# Everybody needs a cheerleader to get a kidney transplant: a qualitative study of the patient barriers and facilitators to kidney transplantation in the Southeastern United States

**DOI:** 10.1186/s12882-016-0326-3

**Published:** 2016-07-30

**Authors:** Teri Browne, Ahinee Amamoo, Rachel E. Patzer, Jenna Krisher, Henry Well, Jennifer Gander, Stephen O. Pastan

**Affiliations:** 1 College of Social Work, University of South Carolina, 12 Hamilton, Columbia, SC 29208 USA; 2 Southeastern Kidney Council, 1000 St. Albans Drive, Suite 270, Raleigh, NC 27609 USA; 3 School of Medicine, Emory University, 101 Woodruff Circle, 5105 WMB, Atlanta, GA 30322 USA; 4 National Kidney Foundation, 508 Hampton Street, Columbia, SC 29201 USA

## Abstract

**Background:**

Kidney transplantation (KTx) disparity is a significant problem in the United States, particularly in the Southeastern region. In response to this phenomenon, the Southeastern Kidney Transplant Coalition was created in 2011 to increase the KTx rate, and to reduce disparities in access to transplantation in the Southeast, by identifying and reducing barriers in the transplant process.

**Methods:**

To determine perceived barriers and facilitators to KTx that dialysis patients in this region experience, we conducted three focus groups with 40 total patients in Georgia, North Carolina, and South Carolina.

**Results:**

We identified two novel themes specific to Southeastern dialysis patients that describe the major barriers and facilitators to kidney transplantation: dialysis center approaches to patient education about KTx, and dialysis center advocacy and encouragement for KTx. In addition, themes related to barriers and facilitators of KTx were evident that were previously mentioned in the literature such as age, fear, knowing other patients with good or bad experiences with KTx, distrust of the KTx process equity, financial concerns and medical barriers.

**Conclusions:**

Dialysis providers are encouraged to enhance their delivery of information and active assistance to underserved patients related to KTx.

**Electronic supplementary material:**

The online version of this article (doi:10.1186/s12882-016-0326-3) contains supplementary material, which is available to authorized users.

## Background

Despite the known benefits of kidney transplantation (KTx), some patients with End Stage Renal Disease (ESRD) who are medically eligible, adequately insured, and interested in transplant struggle to get a transplant [1–4]. This is particularly the case for underserved patients, including minorities, older adults, and women [5–7]. In the Southeastern United States, kidney transplant disparity is particularly significant among African American patients who comprise 67 % of the prevalent ESRD population; yet, the KTx rate for this population is the lowest in the country [8–10]. In response to this phenomenon, in 2010 the Southeastern Kidney Transplant Coalition was created by the Southeastern Kidney Council (End Stage Renal Disease Network 6), to increase the KTx rate in this region by identifying and reducing barriers in the KTx process. This coalition is composed of more than 40 interdisciplinary members of the kidney community representing patients, dialysis companies, dialysis and KTx team members, transplant centers, organ procurement organizations, kidney patient advocacy organizations, and other stakeholders [11] and began work to identify KTx barriers through literature reviews, stakeholder meetings and strategic planning.

Previous research across the US has linked inadequate patient education and information [12, 13], poor provider communication [14] or education about KTx [15, 16], patient difficulty navigating the pathway to KTx [7], and environmental and social factors [8, 17–19] as barriers to underserved patients receiving a KTx. As robust as the existing literature is in this area, there has not yet been any qualitative exploration of patient-identified barriers of kidney transplant in the region that has the greatest kidney transplant disparity in the country -- the Southeast. The composition of the patient population in this region is significantly different than the rest of the United States, with more African Americans and a higher prevalence of ESRD combined with greater rates of poverty, chronic illness and the fewest KTx centers in the country [11]. To inform the coalition’s patient-centered intervention planning and address the gap in the literature related to patient-centered insight about KTx in the Southeastern United States, we conducted three kidney patient focus groups in Georgia, North Carolina, and South Carolina (the three states comprising ESRD Network 6) to get patient feedback about their perceived barriers and facilitators to KTx.

## Methods

### Study design

In 2012, we conducted a qualitative study of patients on dialysis in Atlanta, Georgia; Columbia, South Carolina; and Greensboro, North Carolina using focus group methodology [20, 21] that has been previously used with patients with kidney disease [22, 23]. A convenience sample of forty total adult participants was recruited from attendees at an educational event hosted by the National Kidney Foundation in each of those cities. As this was a convenience sample (i.e. event participants were asked if they wanted to participate in the group), the inclusionary criteria for participants were that they were on dialysis and spoke English. Each focus group was 90 min in length, and participants also completed an optional brief anonymous survey to determine patient demographics and interest in KTx. The focus groups were anonymous, and patients did not share their names or identify the dialysis units where they received treatment.

The group moderators used an interview guide [20] (see Additional file 1) the Coalition members created which included probes about patient interest in KTx as a treatment modality, concerns or barriers patients have about getting a KTx, and facilitators or ways that dialysis team members can help patients get KTx. Two authors facilitated each group. The moderators audiotaped and trained research assistants transcribed all group discussions.

### Participants

Patients were eligible for the focus group if they spoke English, were on dialysis, and were willing and able to participate in the session. Forty total patients participated in the three focus groups in Georgia (*n* = 12), North Carolina (*n* = 9) and South Carolina (*n* = 9) and received a $20 gift card as incentive.

### Data analysis

Nine independent coders conducted manual content analysis of the group transcripts by utilizing qualitative analysis techniques [20, 21, 24–28]. In addition to the authors, five trained research assistants coded all transcripts independently. The coders individually examined the details of the transcripts, initial coded the content for patterns, identified the major categories of the codes, and created relevant themes found in the transcripts [29] through the use of a constant comparison method [27], reviewing every line in the transcripts. After this initial analysis, the nine coders compared, discussed, and synthesized the findings and created a list of the final themes based on group consensus.

## Results

Twenty-nine participants (73 %) completed a brief optional demographic survey following the focus group sessions (see Table 1). Fourteen (48 %) of these were men and 25 (86 %) were African American. Of the 26 patients who noted the length of time they had been on dialysis, the majority (46 %) were on dialysis for more than 2 years. Seven patients (27 %) were on dialysis for 1–2 years and 27 % were on dialysis for less than 1 year. Almost half (48 %) of the participants who provided their age (*n* = 27) were between the ages of 50–59, and 41 % were 40–49 years old. Of the 29 participants who filled out the survey, 76 % indicated that they were interested in a KTx, and 66 % noted that they have not been referred to a KTx center for evaluation. Two themes unique to these dialysis patients that emerged from these focus groups were dialysis center approaches to patient education about KTx and dialysis center encouragement and assistance for KTx. In all three of the focus groups, themes related to barriers and facilitators of KTx were also discovered that were previously mentioned in the literature such as age, fear, knowing other patients with good or bad experiences with KTx [1], distrust of the KTx process equity [30, 31], financial concerns [2] and medical barriers [31].

**Table 1:** Characteristics of focus group participants who completed optional survey

Participant characteristics	n (%)
Age	
18–29 years	1 (4)
40–49 years	11 (41)
50–59 years	13 (48)
60–69 years	2 (7)
Female	15 (52)
Race	
Caucasian	2 (7)
Black/African American	25 (86)
Other	2 (7)
Dialysis Vintage	
0–6 months	3 (12)
6–12 months	4 (15)
1–2 years	7 (27)
> 2 years	12 (46)
Interested in persuing kidney transplantation as a treatment option for kidney disease	
Yes	22 (76)
No	4 (14)
Maybe	3 (10)
Referred to transplant center for evaluation	
Yes	10 (34)
No	19 (66)

### Dialysis center approaches to patient education about kidney transplantation

A major barrier and facilitator to KTx that was mentioned several times in each focus group was information (or lack thereof) patients received from their dialysis team members about transplant, and how to get a KTx. The majority of patients agreed that they do not receive enough information about KTx from their dialysis teams, or that the information they do receive was often not helpful or pro forma. When asked about their current knowledge about KTx, one patient, who shared that he was on dialysis for 2 year, stated, “I just didn’t know…. I’m just not learning.” In a different focus group, a patient said that “we stay in a state of confusion, we don’t understand.” One patient in Georgia shared:

*I’m a new dialysis patient and I know absolutely nothing about the transplant process or anything. At the clinic that I attend it’s kind of hush-hush. They don’t say anything about it.*

Patients in all three states claimed they felt overwhelmed with information that dialysis teams give patients, and that information about KTx is not presented in a meaningful way, as one patient in South Carolina expressed:

*Being sick we don’t really think as well as we used to, and they hit you with a lot of terms at once and all this. What we really need is someone who knows the system inside and out, they understand all the pieces and parts, to sit down with us and say you’ve got these 7 things done and these 7 things are not done…We don’t get that and…they’re withholding information. They just don’t give it to us.*

Patients also claimed they felt overwhelmed with information or given information in a perfunctory manner, as one patient in North Carolina stated:

*They show us a tape every year [about KTx] when we sit in the little lobby, and they pop that little tape in, and they say you need to watch this, and we don’t watch it because we sit in the lobby and talk. And then we go in the back [to the dialysis unit], and then they say, sign this piece of paper saying you saw the video. So we sign it. [They ask] “Did you understand all that you saw”? Yes we did. And you know, we don’t, we sitting out there talking about what we cooked, what the kids did, all the things but what’s actually going on on that video screen. We never saw it. But we lie when we get in the back and say yes, I did watch it. And they know that we didn’t watch it either.*

Patients in all three groups agreed that they need more, and better, information and education from their dialysis centers about KTx. Patients verbalized an appreciation for information about KTx from their interdisciplinary dialysis teams. One patient summed up this sentiment by stating: “If you’re educated, you can make it through.”

### Dialysis center encouragement and assistance

The second novel theme from these focus groups is the importance of advocacy and encouragement from dialysis team members related to KTx. As one individual summarized: “everybody needs a cheerleader” [to get a KTx]. Many patients in all three groups lamented that some dialysis professionals’ behaviors were a discouragement to pursuing KTx. As one participant shared:

*When we started with the nephrologist, when we were first diagnosed with renal failure, and we went into the office and saw the nephrologist, we should have been given options then…when I came in to see her, she never introduced herself, she walked in the room and she said…we’re going to put you in the hospital today, we’re going to do a biopsy, we’re going to do, she said like twenty things. I said well…who are you? She said I’m Dr. so and so, I said interesting, I’m not doing any of that. You gonna need to rewind, go back out the door, come back in, tell me who you are, and then after you tell me who you are, let’s discuss my options. Because you not giving me a choice, so if you don’t give me a choice, well that doesn’t work for me, I don’t know about anybody else, I shut down. If I don’t have no hope, what’s the purpose in us doing this? If you told me I have the ability to have a transplant, and showed me my option for transplant then, oh we could have probably had a wonderful relationship, but to this day she don’t like me because it was ugly. When you walk in the door and you’re not giving your people the option* [of KTx]*, and I’m, well anyway… when you are given no hope, transplant is hope. We’re not given that hope.*

Conversely, patients in all three groups also identified characteristics of helpful dialysis professionals. They explained that having someone on the dialysis team who was empathetic, informative, and a patient advocate was essential in navigating the pathway to KTx. There was no consensus as to who on the dialysis team was most helpful—some patients said it was their physician; others stated it was their social worker, nurse, or patient care technician. Several different patients talked about having a designated team member who was responsible for helping patients get a KTx, and how that was very helpful. One patient explained how his nurse fulfills this role, stating, “I talk to her about it, you know, getting on the transplant list and what I had to do and she talked me through it and we went through the process.”

Another patient described how he felt because he did not have such an individual in his dialysis unit:

[It would be helpful]*” just to have someone just inform you and talk to you about different options or, you know, just try and encourage you. You know, my first few years on dialysis was a disaster. I didn’t have nobody to talk to me, I didn’t have nobody, you know, I had my family but they don’t know.”*

Many patients in each group expressed the belief that being a good self-advocate, and successfully managing their own care was a significant facilitator in pursuing a KTx. One patient summed up this theme by stating: “you have to be your own quarterback” when asked what patients could do to be successful in getting a KTx. The consensus from all three focus groups was that their dialysis centers needed to be more encouraging about KTx and give directed assistance so that patients can pursue KTx to augment patients’ self advocacy.

### Other barriers

In all three of the focus groups, themes related to barriers and facilitators of KTx were discovered that were previously mentioned in the literature. A major component of these other barriers involved patient beliefs about distrusting the entire KTx process. When asked who they thought got kidney transplants, the majority of patients in the three groups responded that they believed individuals who were white and had a higher income, education, and status were more likely to get a KTx. This is exemplified by the following quotes from three different patients: “*I always say the doctor don’t treat you right if you don’t have the right kind of insurance*,” “*I believe the one gets it* [KTx] *is who has the most money to pay for it*,” and:

*I think it has to do a lot with income, and not only just income, this is just something that I saw, you know on TV, you know, I don’t know if it’s true or not, but just based on some things that the individual is doing based on statistics and then there was a, uh, I don’t know if he was a football player or baseball player just recently diagnosed and you know, (snapping of the fingers) just like that, at the drop of a hat, he was able to get a transplant within a week*.

## Discussion

This qualitative study allowed patients on dialysis in Georgia, North Carolina, and South Carolina to identify the barriers and facilitators to KTx that were most salient to them to inform further patient-centered research and help individuals in this region get a KTx. Although some of the findings from this study are similar to previous work, the participants in this study identified novel areas for interventions to promote or discourage KTx that can be useful in this region and beyond. This study is also unique in targeting patients in the Southeast, where KTx rates are the lowest in the nation, an essential first step in informing relevant interventions.

The current Centers for Medicaid and Medicare (CMS) Conditions for Coverage for End-Stage Renal Disease Facilities clearly mandate that all dialysis facilities should be providing patients with information and assistance related to KTx [32]. Our finding that 76 % of focus group participants who completed their demographic forms were interested in KTx, but only 34 % had been referred to a transplant center for evaluation, is consistent with previous research conducted after the 2008 implementation of the Conditions for Coverage that suggests that despite these regulations, many patients on dialysis (particularly underserved populations) in the United States are interested in getting a kidney transplant but lack necessary information about kidney transplantation and how to get a kidney transplant [1, 2, 6, 33–37]. This qualitative study may help elucidate why this is happening in some dialysis units.

This work also suggests that providers can improve their patient-centered delivery of care related to KTx assistance. As the emphasis on patient-centered care [38–40] and shared decision making [38, 41] increases in health delivery systems in ESRD and beyond, dialysis providers may find some of the suggestions from the patients in our study helpful in their own quality assurance performance initiative projects, and researchers can build upon these findings in future intervention research. For example, dialysis units can provide patients with repeated and meaningful information and assistance related to KTx, and make sure that patients understand the education they receive. Furthermore, they may want to encourage patients’ abilities to better self-manage [40] their ESRD care and KTx pursuit so that patients can be their “own quarterbacks” and advocate for themselves more effectively. The patients who participated in these focus groups made it clear that it is not enough for dialysis providers to simply give out information about KTx. Patients want meaningful and active assistance with the transplant process. It is also critical to address patients’ distrust of the transplant process in order to help the most underserved patients.

There are several limitations to the study findings. Inherently, focus groups are limited by small sample size, discussions can be difficult to control, participants may not contribute equally to the discussion and the group moderator may impact participant responses because of the way the questions are delivered. In our study, the sample size is intentionally small in order to explore in-depth individual feelings and beliefs. We controlled the group and participation of all group members by only allowing one person to speak at a time, directly encouraging all members to participate, and “calling on” different patients to contribute so that a few patients did not dominate the conversation. We also only used questions from our interview guide to insure that the moderators did not influence the patient responses. Despite the possible limitations of focus groups, there are also many advantages to this research methodology. Focus groups allow for an in-depth exploration of opinion that is not possible in quantitative research and may be more inclusive as participants do not have to have the literacy and numeracy skills necessary to complete surveys or other methods of data collection.

The Georgia focus group with 12 patients is slightly larger than the 6–10 participants recommended [21], however that group did have two facilitators directing the conversation and all patients had an opportunity to share their feelings about the interview questions. Because the groups were entirely anonymous and no patient names were revealed or used, it may be that group rapport was diminished however each group had robust discussions involving all attendees. This research was conducted with a convenience sample of patients in only three states; these participants might not be typical patients on dialysis, in that they were motivated to attend a patient education event and participate in the focus group. In addition, as only one focus group was conducted in these three states, the patients in this study may not be representative of all patients in their state. Therefore, as with any qualitative research, the findings have limited generalizability even within the three states where this research occurred.

However, this information may be particularly useful for the Southeastern region of the US, which has dialysis facilities with the lowest KTx rates in the nation and cares for the most underserved patients [42]. As this study was entirely anonymous and the investigators were not privy to the patient medical records, we have no way of knowing if the focus group patients would even be medically eligible and suitable for a KTx. Furthermore, our convenience sample was not exactly reflective of the demographic composition of Network 6 (e.g., in Network 6, 55 % prevalent patients are men and 67 % are African American. In this study 48 % of respondents who completed the survey were men and 86 % African American). However, we are encouraged that the findings are robust as they are congruent with previous quantitative research across the country and suggest that: (a) there is a sizeable portion of patients on dialysis who mistakenly believe that they are active on a KTx list, when in fact they are not [1, 33–37] and (b) that dialysis providers may not be effectively communicating with patients about KTx [33].

This study complements the growing body of international qualitative research with kidney disease and transplant patients, including studies that examine the development of patient education materials about KTx and other treatment options [43, 44]; issues related to living kidney donation [45, 46]; patient understanding of increased-risk donor kidneys [22, 47]; the experience of kidney transplant rejection [48]; donor-recipient relationships [49]; the kidney disease trajectory [23]; kidney disease research priorities [50] and kidney treatment options [13]. This is the first study to specifically explore the patient-identified facilitators and barriers to seeking a KTx in the Southeastern United States.

Patient perceived barriers and facilitators to KTx are just one attribute contributing to KTx rates, in addition to other patient factors (i.e. medical and financial eligibility) as well as dialysis facility, geographical, and transplant center factors. These focus groups provide insight that can inform larger scale studies. Accordingly, the Southeastern Kidney Transplant Coalition has surveyed all dialysis units in the Network about their KTx practices [51, 52] and continues to conceptualize KTx referral success as dependent on multiple factors.

## Conclusions

To our knowledge, this is the first study to specifically explore the patient-identified facilitators and barriers to seeking a KTx in the Southeastern United States. Because this study was conducted in the area with the highest concentration of African American patients receiving dialysis, the study may also be helpful to other ESRD networks that are now mandated by CMS to attend to ESRD disparities, including the promotion of KTx [5]. The most important result of qualitative work such as this may be to remind the ESRD community, in the words of one of the focus group attendees, that “everybody needs a cheerleader” to get a KTx.

## Additional file

Additional file 1: Focus Group Interview Guide. This is the interview guide used for all of the focus groups in this study. (DOCX 14 kb)
