# Supplementary material for: Everybody needs a cheerleader to get a kidney transplant: a qualitative study of the patient barriers and facilitators to kidney transplantation in the Southeastern United States
Source: BMC Nephrol. 2016 Jul 30;17:108. doi: 10.1186/s12882-016-0326-3 (PMC4967312; doi:10.1186/s12882-016-0326-3)
Supplement: Additional file 1: — Focus Group Interview Guide. This is the interview guide used for all of the focus groups in this study. (DOCX 14 kb) [file 12882_2016_326_MOESM1_ESM.docx]

**Interview Guide**

**Southeastern Kidney Transplant Coalition Patient Focus Groups**

Theme 1: Patient interest / barriers

1. Tell me about your interest in getting a kidney transplant.
2. What is your biggest concern about getting a kidney transplant?
3. What were some of the specific challenges or obstacles you think you will experience with regard to trying to get a kidney transplant?
4. Who and/or what do you think can help you most to overcome (manage) the challenges or obstacles associated with getting a kidney transplant?
5. What does your family think about you getting a transplant?

Theme 2: Facility role/encouragement

1. Does your kidney doctor encourage you to get a transplant?
2. Does your dialysis facility staff encourage you to get a transplant?
3. Who in your facility is the person who mostly tells you about kidney transplant?
4. How often do you talk to that person about transplant?
5. When your dialysis team member talks with you about getting a kidney transplant, does she or he make sure that you’ve had all of your questions answered?
6. What do you think are the characteristics of people who are most likely to get transplanted?
7. What are the characteristics of people who are LEAST likely to get transplanted?
8. Tell me what your dialysis team can do to help you get a kidney transplant.
9. What else would help you better get a kidney transplant?

Theme 3: Patient Knowledge/Understanding

1. Tell me what you have learned about how to get a kidney transplant.

PROBES:

1. What is your biggest concern about getting a kidney transplant?
2. What were some of the specific challenges or obstacles you experienced with regard to getting a kidney transplant?
3. How did you overcome (manage) the challenges or obstacles associated with getting a kidney transplant?
4. What prevented you from overcoming (managing) the challenges or obstacles associated with getting a kidney transplant?
